# Crystal structure of *trans*-di­aqua­(1,4,8,11-tetra­aza­undeca­ne)nickel(II) bis­(pyridine-2,6-di­carboxyl­ato)nickel(II)

**DOI:** 10.1107/S2056989021011178

**Published:** 2021-10-29

**Authors:** Irina L. Andriichuk, Liudmyla V. Tsymbal, Vladimir B. Arion, Yaroslaw D. Lampeka

**Affiliations:** a L. V. Pisarzhevskii Institute of Physical Chemistry of the National Academy of Sciences of Ukraine, Prospekt Nauki 31, 03028 Kiev, Ukraine; b Institute of Inorganic Chemistry of the University of Vienna, Wahringer Str., 42, 1090 Vienna, Austria

**Keywords:** crystal structure, cyclam, nickel, pyridine-2,6-di­carboxyl­ate, hydrogen bonds

## Abstract

The asymmetric unit of the title compound, *trans*-di­aqua­(1,4,8,11-tetra­aza­undecane-κ^4^ *N* ^1^,*N* ^4^,*N* ^8^,*N* ^11^)nickel(II) bis­(pyridine-2,6-di­carboxyl­ato-κ^3^ *O* ^2^,*N*,*O* ^6^)nickel(II) {[Ni(*L*)(H_2_O)_2_][Ni(pdc)_2_] where *L* = 1,4,8,11-tetra­aza­undecane (C_7_H_20_N_4_) and pdc = the dianion of pyridine-2,6-di­carb­oxy­lic acid (C_7_H_3_NO_4_^2−^)} consists of an [Ni(*L*)(H_2_O)_2_]^2+^ complex cation and a [Ni(pdc)_2_]^2–^ anion. The metal ion in the cation is coordinated by the four N atoms of the tetra­amine ligand and the mutually *trans* O atoms of the water mol­ecules in a tetra­gonally elongated octa­hedral geometry with the average equatorial Ni—N bond length slightly shorter than the average axial Ni—O bond [2.087 (4) *versus* 2.128 (4) Å]. The ligand *L* adopts its energetically favored conformation with five-membered and six-membered chelate rings in *gauche* and *chair* conformations, respectively. In the complex anion, the Ni^II^ ion is coordinated by the two tridentate pdc^2–^ ligands *via* their carboxyl­ate and nitro­gen atom donors in a distorted octa­hedral *trans*-NiO_4_N_2_ geometry with nearly orthogonal orientation of the planes defining the carboxyl­ate rings and the average Ni—N bond length [1.965 (4) Å] shorter than the average Ni—O bond distance [2.113 (7) Å]. In the crystal, the NH donor groups of the tetra­amine, the carb­oxy­lic groups of the pdc^2–^ anion and the coordinated water mol­ecules are involved in numerous N—H⋯O and O—H⋯O hydrogen bonds, leading to electroneutral sheets oriented parallel to the (001) plane.

## Abstract 2

The coordination polyhedra of the nickel(II) ions of the title compound in the complex cation and the anion, *viz*., *trans*-NiN_4_O_2_ and *trans*-NiO_4_N_2_, are distorted octa­hedra. In the crystal, the donor groups of the tetra­amine and the coordinated water mol­ecules and the carboxyl­ate groups of the pyridine-2,6-di­carboxyl­ate anions are involved in numerous N—H⋯O and O—H⋯O hydrogen bonds, thereby forming sheets of ions lying parallel to the (001) plane.

## Chemical context

Crystalline coordination polymers possessing permanent porosity (metal–organic frameworks, MOFs) are of enormous current inter­est because of their potential for applications in different areas including gas storage, separation, catalysis, *etc*. (MacGillivray & Lukehart, 2014 ▸; Kaskel, 2016 ▸). Nickel(II) complexes of the 14-membered macrocyclic tetra­amine ligands, in particular of cyclam and its C-alkyl­ated derivatives (cyclam = 1,4,8,11-tetra­aza­cyclo­tetra­decane, C_10_H_24_N_4_), are widely used as metal-containing building units for the construction of MOFs (Lampeka & Tsymbal, 2004 ▸; Suh & Moon, 2007 ▸; Suh *et al.*, 2012 ▸; Stackhouse & Ma, 2018 ▸; Lee & Moon, 2018 ▸). At the same time, nickel(II) complexes of 1,4,8,11-tetra­aza­undecane (C_7_H_20_N_4_; *L*) – the closest open-chain analogue of cyclam – are rarely utilized for the construction of MOFs and only a few examples of coordin­ation polymers formed by the [Ni(*L*)]^2+^ cation with azide (Escuer *et al.*, 1993 ▸), cyanide (Koo *et al.*, 2003 ▸), and cyano­metalate (Koo *et al.*, 2003 ▸; Shek *et al.*, 2005 ▸; Talukder *et al.*, 2012 ▸; Ni *et al.*, 2014 ▸) bridging anions have been characterized by single-crystal X-ray diffraction. 
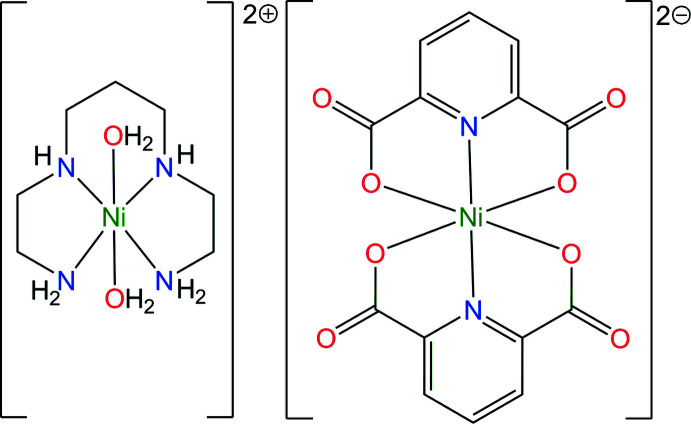


Multidentate aromatic carboxyl­ates are known as the most common linkers in MOFs (Rao *et al.*, 2004 ▸). Although the bridging properties of one of the simplest representative of this class of compounds, 1,3-benzene­dicarboxyl­ate, with macrocyclic nickel(II) cations are well studied (see, for example, Tsymbal *et al.*, 2021 ▸), coordination polymers based on its structural analogue, pyridine-2,6-di­carboxyl­ate (C_7_H_3_NO_4_^2–^; pdc^2–^), are confined to a sole example (Choi *et al.*, 2003 ▸). Inter­estingly, an attempt to prepare a coordination polymer containing the [Ni(cyclam)]^2+^ cation with pdc^2–^ led to the ionic product [Ni(cyclam)(H_2_O)_2_][Ni(pdc)_2_]·2.5H_2_O due to sequestering of the metal ion from the cavity of the macrocycle by this chelating ligand (Park *et al.*, 2007 ▸).

As part of our research on MOFs formed by nickel(II) tetra­aza cations and aromatic carboxyl­ates, we report here the synthesis and crystal structure of the product of the reaction of [Ni(*L*)]^2+^ with pdc^2–^, namely [*trans*-di­aqua­(1,4,8,11-tetra­aza­undecane-*k* ^4^ *N* ^1^ *N* ^4^ *N* ^8^ *N* ^11^)nickel(II)][bis­(pyridine-2,6-di­carb­oxy­lato-*κ* ^3^ *N*,*O*,*O*)nickel(II)], [Ni(*L*)(H_2_O)_2_][Ni(pdc)_2_], **I**. Similar to the reaction of pyridine-2,6-di­carboxyl­ate with the [Ni(cyclam)]^2+^ cation, the formation of the title compound is explained by the sequestering of the metal ion from the starting cation with the formation of the [Ni(pdc)_2_]^2–^ anion. Additionally, to the best of our knowledge, the structure of the [*trans*-di­aqua­(1,4,8,11-tetra­aza­undeca­ne)nickel(II)] moiety has not previously been reported in the literature.

## Structural commentary

**Figure 1:**
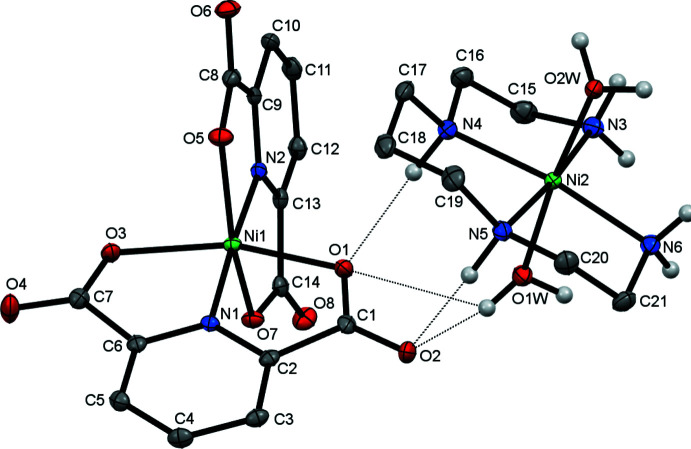
View of the mol­ecular structure of **I**, showing the partial atom-labeling scheme, with displacement ellipsoids drawn at the 40% probability level. C-bound H atoms are omitted for clarity. Hydrogen-bonding inter­actions are shown as dotted lines.

The mol­ecular structure of the title compound **I** is shown in Fig. 1 ▸. Atom Ni1 is coordinated by the two tridentate pdc^2–^ ligands *via* their carboxyl­ate and nitro­gen donors, resulting in the formation of the [Ni(pdc)_2_]^2–^ divalent anion, which is charge-balanced by the [Ni(*L*)(H_2_O)_2_]^2+^ divalent cation formed by atom Ni2.

The coordination polyhedron of Ni1^II^ in the complex anion ion can be described as a tetra­gonally compressed *trans*-NiO_4_N_2_ octa­hedron with the Ni—N bond lengths [average value 1.965 (4) Å] shorter than the Ni—O ones [average value 2.113 (7) Å] (Table 1 ▸). Another source of distortion is the alternating displacement (by *ca* 0.43 Å) of the coordinated oxygen atoms of deprotonated carb­oxy­lic groups from the mean equatorial plane formed by the four oxygen atoms. The values of the bite angles in the five-membered chelate rings in the complex anion are very similar (Table 1 ▸). The pdc^2–^ carboxyl­ate rings are oriented nearly orthogonally with an angle of 81.5 (3)° between their mean planes.

The Ni2^II^ ion in the complex cation is coordinated by the four N atoms of the ligand *L* and the mutually *trans* O atoms of the water mol­ecules in a tetra­gonally elongated *trans*-NiN_4_O_2_ octa­hedral geometry with the average equatorial Ni—N bond length slightly shorter than the average axial Ni—O bond [2.087 (4) and 2.128 (4) Å, respectively (Table 1 ▸)]. The ligand *L* in **I** adopts its energetically favored conformation with the five-membered and six-membered chelate rings in *gauche* and *chair* conformations, respectively, which resemble the *trans*-III configuration usually observed in cyclam complexes (Bosnich *et al.*, 1965 ▸). This conformation is also characteristic of the macrocyclic ligand in [Ni(cyclam)(H_2_O)_2_]^2+^ (Park *et al.*, 2007 ▸), although the bite angles in the five-membered (85.54°) and six-membered (94.46°) chelate rings are correspondingly larger and smaller compared to those in **I** (Table 1 ▸).

## Supra­molecular features

**Figure 2:**
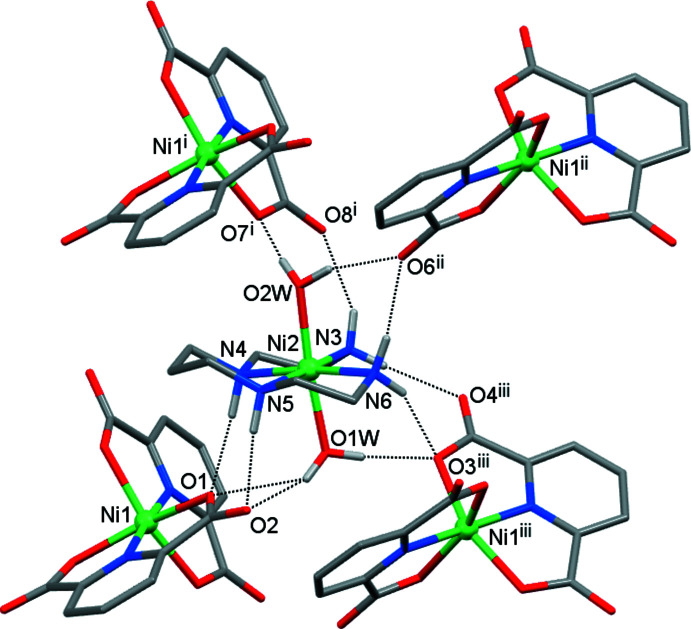
Nearest surroundings of the cation in **I** formed by hydrogen bonding (dotted lines). [Symmetry codes: (i) *x* − 1, *y*, *z*; (ii) −*x* + 1, *y* + 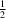 , −*z* + 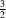 ; (iii) −*x* + 2, *y* + 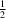 , −*z* + 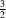 .]

**Figure 4:**
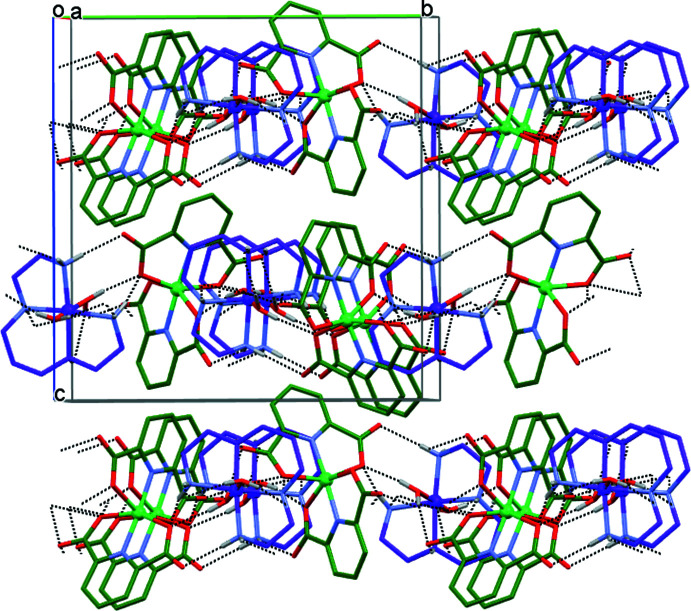
Electroneutral sheets of the complex ions in **I** parallel to the (001) plane. C-bound H atoms are omitted for clarity. C atoms of the cation and anion are shown in purple and green, respectively. Hydrogen bonds are shown as dotted lines.

The crystals of **I** are composed of [Ni(*L*)(H_2_O)_2_]^2+^ complex cations and [Ni(pdc)_2_]^2–^ anions connected by numerous hydrogen bonds (Table 2 ▸). Each ion is surrounded by four counter-ions (Figs. 2 ▸ and 3 ▸); the cation acts as the hydrogen-bond donor due to the presence of the N—H fragments of amino groups and the O—H groups of coordinated water mol­ecules, while the anion displays proton-acceptor properties because of the availability of the carb­oxy­lic groups. These aggregates are further arranged into two-dimensional sheets oriented parallel to the (001) plane (Fig. 4 ▸). There are no hydrogen-bonding contacts between the sheets, and the three-dimensional coherence of the crystal is provided by van der Waals inter­actions.

## Database survey

A search of the Cambridge Structural Database (CSD, version 5.42, last update February 2021; Groom *et al.*, 2016 ▸) indicated that no compounds containing the [Ni(*L*)(H_2_O)_2_]^2+^ cation have been structurally characterized to date, the closest analogue being the complex [Ni(*L*)(H_2_O)(Cl)]Cl (refcode UMOFEH; Oblezov *et al.*, 2003 ▸). In general, the geometrical parameters of both cations in these compounds are similar, although the Ni—O bond length in the latter is longer (2.182 Å), probably because of the *trans* influence of the chloride ligand.

As far as the structures of the cations in the compounds with the same bis­(pyridine-2,6-di­carboxyl­ato)-nickel(II) anion are concerned, {[Ni(*L*)(H_2_O)_2_]^2+^ in **I** and [Ni(cyclam)(H_2_O)_2_]^2+^ in TICJEV (Park *et al.*, 2007 ▸)}, a higher tetra­gonal distortion of the coordination polyhedron in the latter case [average Ni—N bond length of 2.068 (6) Å and Ni—O bond length of 2.152 Å] should be mentioned, which can be explained by the stronger *cis* influence of the macrocyclic ligand compared to the non-cyclic one (Yatsimirskii & Lampeka, 1985 ▸).

## Synthesis and crystallization

All chemicals and solvents used in this work were purchased from Sigma–Aldrich and used without further purification. The complex [Ni(*L*)](ClO_4_)_2_ was prepared by mixing equimolar amount of *L* and nickel perchlorate hexa­hydrate in ethanol. The title compound **I** was prepared as follows. A solution of [Ni(*L*)](ClO_4_)_2_ (11 mg, 0.026 mmol) in 1 ml of DMF was added to 0.4 ml of an aqueous solution of Na_2_(pdc) (2.7 mg, 0.013 mmol). Blue crystals formed in a day, which were filtered off, washed with diethyl ether and dried in air. Yield: 1.3 mg (15.5%). Analysis calculated for C_21_H_30_N_6_Ni_2_O_10_: C 39.17, H 4.66, N 13.06%. Found: C 39.04, H 5.0, N 13.21%. Single crystals of **I** suitable for X-ray diffraction analysis were selected from the sample resulting from the synthesis.

**Safety note**: Perchlorate salts of metal complexes are potentially explosive and should be handled with care.

## Refinement

Crystal data, data collection and structure refinement details are summarized in Table 3 ▸. H atoms in **I** were placed in geometrically idealized positions and constrained to ride on their parent atoms, with C—H distances of 0.95 (ring H atoms) or 0.99 Å (aliphatic H atoms), N—H distances of 0.91 (primary amino groups) or 1.00 Å (secondary amino­groups) with *U*_iso_(H) values of 1.2*U*_eq_ of the parent atoms. Water H atoms were positioned geometrically (O—H = 0.71–0.85 Å) and refined as riding with *U*_iso_(H) = 1.5*U*_eq_(O).

## Supplementary Material

Crystal structure: contains datablock(s) I. DOI: 10.1107/S2056989021011178/hb7995sup1.cif

Structure factors: contains datablock(s) I. DOI: 10.1107/S2056989021011178/hb7995Isup2.hkl

CCDC reference: 2115829

Additional supporting information:  crystallographic information; 3D view; checkCIF report

## Figures and Tables

**Figure 3:**
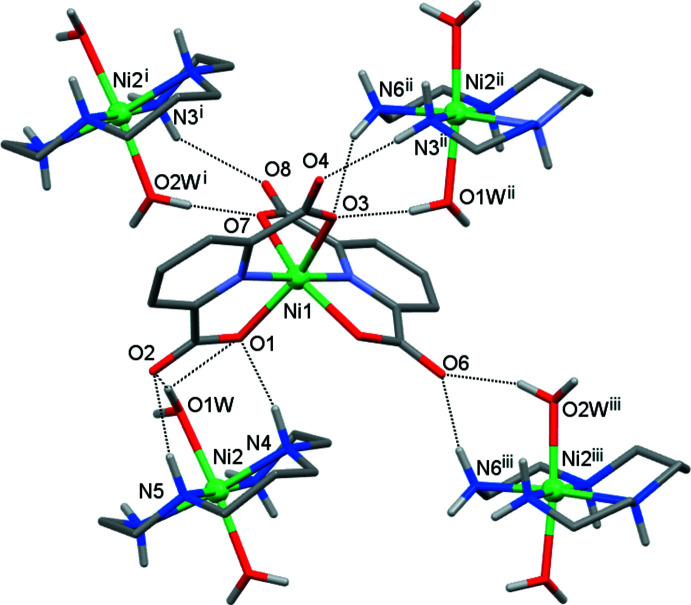
Nearest surroundings of the anion in **I** formed by hydrogen bonding (dotted lines). [Symmetry codes: (i) *x* + 1, *y*, *z*; (ii) −*x* + 2, *y* − 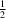 , −*z* + 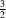 ; (iii) −*x* + 1, *y* − 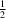 , −*z* + 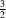

**Table 1:** Selected geometric parameters (Å, °)

Ni1—O1	2.099 (2)	Ni2—O1*W*	2.131 (2)
Ni1—O3	2.109 (2)	Ni2—O2*W*	2.124 (2)
Ni1—O5	2.111 (2)	Ni2—N3	2.074 (2)
Ni1—O7	2.1343 (19)	Ni2—N4	2.088 (2)
Ni1—N1	1.961 (2)	Ni2—N5	2.095 (2)
Ni1—N2	1.969 (2)	Ni2—N6	2.089 (3)
			
O1—Ni1—O3	156.79 (8)	O2*W*—Ni2—O1*W*	174.88 (8)
O1—Ni1—O5	95.74 (8)	N3—Ni2—O1*W*	86.26 (9)
O1—Ni1—O7	89.96 (8)	N3—Ni2—O2*W*	92.25 (9)
O3—Ni1—O5	89.36 (8)	N3—Ni2—N4	84.10 (10)
O3—Ni1—O7	94.68 (8)	N3—Ni2—N5	174.54 (10)
O5—Ni1—O7	155.62 (7)	N3—Ni2—N6	101.01 (10)
N1—Ni1—O1	78.63 (9)	N4—Ni2—O1*W*	87.83 (9)
N1—Ni1—O3	78.19 (9)	N4—Ni2—O2*W*	96.90 (9)
N1—Ni1—O5	105.53 (9)	N4—Ni2—N5	90.45 (10)
N1—Ni1—O7	98.84 (9)	N4—Ni2—N6	172.57 (10)
N1—Ni1—N2	176.06 (10)	N5—Ni2—O1*W*	93.07 (9)
N2—Ni1—O1	99.61 (9)	N5—Ni2—O2*W*	88.86 (9)
N2—Ni1—O3	103.60 (9)	N6—Ni2—O1*W*	87.13 (9)
N2—Ni1—O5	78.10 (9)	N6—Ni2—O2*W*	88.34 (9)
N2—Ni1—O7	77.58 (9)	N6—Ni2—N5	84.36 (10)

**Table 2:** Hydrogen-bond geometry (Å, °)

*D*—H⋯*A*	*D*—H	H⋯*A*	*D*⋯*A*	*D*—H⋯*A*
N3—H3*A*⋯O8^i^	0.91	2.41	3.213 (3)	147
N3—H3*B*⋯O4^ii^	0.91	2.11	3.015 (3)	176
N4—H4*A*⋯O1	1.00	2.07	3.054 (3)	167
N5—H5*A*⋯O2	1.00	2.08	3.054 (3)	163
N6—H6*A*⋯O3^ii^	0.91	2.14	2.986 (3)	154
N6—H6*B*⋯O6^iii^	0.91	2.07	2.943 (3)	160
O1*W*—H1*WA*⋯O1	0.86	2.56	3.088 (3)	121
O1*W*—H1*WA*⋯O2	0.86	2.00	2.795 (3)	154
O1*W*—H1*WB*⋯O3^ii^	0.86	1.91	2.757 (3)	170
O2*W*—H2*WA*⋯O7^i^	0.87	1.80	2.663 (3)	169
O2*W*—H2*WB*⋯O6^iii^	0.87	1.90	2.742 (3)	160

Symmetry codes: (i) x-1, y, z; (ii) -x+2, y+{\script{1\over 2}}, -z+{\script{3\over 2}}; (iii) -x+1, y+{\script{1\over 2}}, -z+{\script{3\over 2}}.

**Table 3:** Experimental details

Crystal data	
Chemical formula	[Ni(C_7_H_20_N_4_)(H_2_O)_2_][Ni(C_7_H_3_NO_4_)_2_]
*M*_r_	643.93
Crystal system, space group	Orthorhombic, *P*2_1_2_1_2_1_
Temperature (K)	100
*a*, *b*, *c* (Å)	9.3219 (6), 16.3211 (10), 16.9483 (8)
*V* (Å^3^)	2578.6 (3)
*Z*	4
Radiation type	Mo *K*α
μ (mm^−1^)	1.53
Crystal size (mm)	0.25 × 0.2 × 0.2

Data collection	
Diffractometer	Bruker APEXII CCD
Absorption correction	Multi-scan (*CrysAlis PRO*; Rigaku OD, 2019 ▸)
*T*_min_, *T*_max_	0.705, 0.737
No. of measured, independent and observed [*I* > 2σ(*I*)] reflections	36128, 4909, 4668
*R*_int_	0.045
(sin θ/λ)_max_ (Å^−1^)	0.610

Refinement	
*R*[*F* ^2^ > 2σ(*F* ^2^)], *wR*(*F* ^2^), *S*	0.021, 0.050, 1.04
No. of reflections	4909
No. of parameters	356
H-atom treatment	H atoms treated by a mixture of independent and constrained refinement
Δρ_max_, Δρ_min_ (e Å^−3^)	0.49, −0.26
Absolute structure	Flack *x* determined using 1953 quotients [(*I* ^+^)−(*I* ^−^)]/[(*I* ^+^)+(*I* ^−^)] (Parsons *et al.*, 2013 ▸)
Absolute structure parameter	−0.010 (4)

Computer programs: *CrysAlis PRO* (Rigaku OD, 2019 ▸), *SHELXT2018/2* (Sheldrick, 2015*a* ▸), *SHELXL2018/3* (Sheldrick, 2015*b* ▸), *Mercury* (Macrae *et al.*, 2020 ▸) and *publCIF* (Westrip, 2010 ▸).

## supplementary crystallographic information

### Crystal data

**Table:**

[Ni(C_7_H_20_N_4_)(H_2_O)_2_][Ni(C_7_H_3_NO_4_)_2_]	*D*_x_ = 1.659 Mg m^−^^3^
*M**_r_* = 643.93	Mo *K*α radiation, λ = 0.71073 Å
Orthorhombic, *P*2_1_2_1_2_1_	Cell parameters from 8341 reflections
*a* = 9.3219 (6) Å	θ = 2.5–25.3°
*b* = 16.3211 (10) Å	µ = 1.53 mm^−^^1^
*c* = 16.9483 (8) Å	*T* = 100 K
*V* = 2578.6 (3) Å^3^	Prism, clear light pink
*Z* = 4	0.25 × 0.2 × 0.2 mm
*F*(000) = 1336	

### Data collection

**Table:**

Bruker APEXII CCD diffractometer	4668 reflections with *I* > 2σ(*I*)
Radiation source: fine-focus sealed tube	*R*_int_ = 0.045
φ and ω scans	θ_max_ = 25.7°, θ_min_ = 2.4°
Absorption correction: multi-scan (CrysAlisPro; Rigaku OD, 2019)	*h* = −11→11
*T*_min_ = 0.705, *T*_max_ = 0.737	*k* = −19→19
36128 measured reflections	*l* = −20→20
4909 independent reflections	

### Refinement

**Table:**

Refinement on *F*^2^	Hydrogen site location: mixed
Least-squares matrix: full	H atoms treated by a mixture of independent and constrained refinement
*R*[*F*^2^ > 2σ(*F*^2^)] = 0.021	*w* = 1/[σ^2^(*F*_o_^2^) + (0.0243*P*)^2^ + 0.7375*P*] where *P* = (*F*_o_^2^ + 2*F*_c_^2^)/3
*wR*(*F*^2^) = 0.050	(Δ/σ)_max_ = 0.002
*S* = 1.04	Δρ_max_ = 0.49 e Å^−^^3^
4909 reflections	Δρ_min_ = −0.26 e Å^−^^3^
356 parameters	Absolute structure: Flack *x* determined using 1953 quotients [(*I*^+^)-(*I*^-^)]/[(*I*^+^)+(*I*^-^)] (Parsons *et al.*, 2013)
0 restraints	Absolute structure parameter: −0.010 (4)
Primary atom site location: dual	

### Special details

**Table:**

Geometry. All esds (except the esd in the dihedral angle between two l.s. planes) are estimated using the full covariance matrix. The cell esds are taken into account individually in the estimation of esds in distances, angles and torsion angles; correlations between esds in cell parameters are only used when they are defined by crystal symmetry. An approximate (isotropic) treatment of cell esds is used for estimating esds involving l.s. planes.

### Fractional atomic coordinates and isotropic or equivalent isotropic displacement parameters (Å^2^)

**Table:**

	*x*	*y*	*z*	*U*_iso_*/*U*_eq_	
Ni1	1.09379 (4)	0.79146 (2)	0.79300 (2)	0.01132 (9)	
O1	0.9734 (2)	0.89973 (12)	0.80207 (12)	0.0156 (4)	
O3	1.2374 (2)	0.69917 (13)	0.82970 (11)	0.0150 (4)	
O2	0.9525 (2)	1.01235 (13)	0.87640 (12)	0.0190 (5)	
O4	1.3661 (3)	0.65997 (13)	0.93534 (13)	0.0250 (5)	
O5	0.9208 (2)	0.71131 (13)	0.81555 (11)	0.0178 (4)	
O7	1.2475 (2)	0.85377 (12)	0.72153 (10)	0.0151 (4)	
O6	0.7669 (2)	0.62760 (13)	0.75366 (12)	0.0208 (5)	
O8	1.2894 (2)	0.88805 (13)	0.59530 (12)	0.0197 (5)	
N1	1.1543 (3)	0.83219 (15)	0.89664 (13)	0.0117 (5)	
N2	1.0390 (3)	0.75735 (15)	0.68573 (13)	0.0124 (5)	
C1	1.0034 (3)	0.94313 (19)	0.86156 (17)	0.0147 (6)	
C2	1.1083 (3)	0.90574 (17)	0.91977 (15)	0.0136 (6)	
C3	1.1561 (3)	0.93967 (19)	0.98988 (17)	0.0160 (6)	
H3	1.127255	0.993102	1.005442	0.019*	
C4	1.2481 (3)	0.89317 (19)	1.03707 (17)	0.0178 (7)	
H4	1.278944	0.913881	1.086623	0.021*	
C5	1.2944 (3)	0.81687 (19)	1.01178 (16)	0.0155 (6)	
H5	1.357733	0.785035	1.043217	0.019*	
C6	1.2463 (3)	0.78819 (18)	0.93978 (16)	0.0136 (6)	
C7	1.2889 (3)	0.70808 (19)	0.90009 (17)	0.0154 (6)	
C8	0.8683 (3)	0.67716 (17)	0.75527 (17)	0.0149 (6)	
C9	0.9355 (3)	0.70262 (18)	0.67704 (17)	0.0131 (6)	
C10	0.8957 (4)	0.67512 (17)	0.60250 (17)	0.0166 (6)	
H10	0.821798	0.635587	0.596314	0.020*	
C11	0.9667 (3)	0.7068 (2)	0.53760 (18)	0.0194 (7)	
H11	0.941943	0.688728	0.486084	0.023*	
C12	1.0745 (3)	0.76525 (18)	0.54766 (16)	0.0164 (6)	
H12	1.123225	0.787779	0.503466	0.020*	
C13	1.1087 (3)	0.78960 (17)	0.62377 (16)	0.0132 (6)	
C14	1.2247 (3)	0.84969 (18)	0.64704 (16)	0.0147 (6)	
Ni2	0.58765 (4)	1.01066 (2)	0.76468 (2)	0.01201 (9)	
O1W	0.8016 (2)	1.04716 (13)	0.73792 (12)	0.0192 (5)	
H1WA	0.869828	1.041746	0.771368	0.031 (10)*	
H1WB	0.797698	1.092906	0.712918	0.040 (11)*	
O2W	0.3684 (2)	0.98471 (13)	0.78767 (12)	0.0172 (4)	
H2WA	0.325706	0.939783	0.772223	0.042 (12)*	
H2WB	0.308376	1.025333	0.781113	0.069 (16)*	
N3	0.5586 (3)	1.00016 (16)	0.64375 (14)	0.0192 (6)	
H3A	0.465077	0.989548	0.632344	0.023*	
H3B	0.584859	1.047317	0.618999	0.023*	
N4	0.6569 (3)	0.88925 (15)	0.75663 (15)	0.0174 (5)	
H4A	0.763467	0.890194	0.763131	0.021*	
N5	0.6255 (3)	1.01005 (15)	0.88651 (13)	0.0163 (5)	
H5A	0.731507	1.013124	0.894920	0.020*	
N6	0.5419 (3)	1.13467 (15)	0.78184 (14)	0.0170 (6)	
H6A	0.586732	1.165700	0.744666	0.020*	
H6B	0.445736	1.143679	0.778488	0.020*	
C15	0.6505 (4)	0.9317 (2)	0.61828 (18)	0.0236 (7)	
H15A	0.752369	0.948818	0.618268	0.028*	
H15B	0.624339	0.914509	0.564135	0.028*	
C16	0.6286 (4)	0.8613 (2)	0.67532 (19)	0.0226 (8)	
H16A	0.528893	0.840840	0.671222	0.027*	
H16B	0.694390	0.815805	0.661796	0.027*	
C17	0.6005 (4)	0.83218 (18)	0.81722 (18)	0.0210 (7)	
H17A	0.639786	0.776760	0.807346	0.025*	
H17B	0.494866	0.829025	0.812244	0.025*	
C18	0.6383 (4)	0.8580 (2)	0.90014 (19)	0.0234 (8)	
H18A	0.743906	0.863351	0.903632	0.028*	
H18B	0.609403	0.813510	0.936547	0.028*	
C19	0.5716 (4)	0.93725 (19)	0.92903 (18)	0.0230 (7)	
H19A	0.466236	0.934035	0.922537	0.028*	
H19B	0.592126	0.943637	0.986010	0.028*	
C20	0.5617 (4)	1.0860 (2)	0.91737 (18)	0.0216 (8)	
H20A	0.601711	1.098266	0.970176	0.026*	
H20B	0.456571	1.079348	0.922625	0.026*	
C21	0.5947 (4)	1.15564 (18)	0.86117 (17)	0.0207 (7)	
H21A	0.547856	1.206507	0.879817	0.025*	
H21B	0.699559	1.165229	0.859354	0.025*	

### Atomic displacement parameters (Å^2^)

**Table:**

	*U*^11^	*U*^22^	*U*^33^	*U*^12^	*U*^13^	*U*^23^
Ni1	0.01194 (18)	0.01160 (18)	0.01043 (16)	−0.00100 (17)	−0.00079 (17)	−0.00127 (13)
O1	0.0139 (10)	0.0152 (11)	0.0177 (10)	0.0013 (9)	−0.0033 (8)	−0.0023 (9)
O3	0.0179 (11)	0.0130 (11)	0.0142 (10)	0.0014 (9)	−0.0025 (9)	−0.0030 (8)
O2	0.0168 (11)	0.0133 (11)	0.0270 (11)	0.0034 (9)	−0.0032 (8)	−0.0013 (9)
O4	0.0324 (14)	0.0204 (13)	0.0222 (11)	0.0110 (10)	−0.0066 (10)	−0.0003 (9)
O5	0.0170 (11)	0.0199 (11)	0.0164 (10)	−0.0050 (11)	0.0015 (9)	−0.0006 (8)
O7	0.0161 (11)	0.0165 (11)	0.0126 (11)	−0.0039 (9)	−0.0008 (8)	−0.0009 (8)
O6	0.0182 (12)	0.0174 (11)	0.0267 (12)	−0.0069 (10)	0.0006 (9)	−0.0020 (9)
O8	0.0209 (12)	0.0198 (12)	0.0185 (11)	−0.0028 (10)	0.0058 (9)	0.0036 (9)
N1	0.0110 (12)	0.0126 (13)	0.0115 (12)	−0.0015 (10)	0.0003 (10)	−0.0001 (10)
N2	0.0113 (12)	0.0116 (13)	0.0141 (12)	0.0025 (10)	−0.0014 (9)	−0.0013 (10)
C1	0.0084 (16)	0.0155 (17)	0.0203 (15)	0.0004 (13)	0.0032 (12)	0.0010 (12)
C2	0.0135 (16)	0.0124 (15)	0.0148 (13)	−0.0028 (13)	0.0030 (12)	0.0004 (11)
C3	0.0179 (16)	0.0148 (17)	0.0153 (14)	−0.0031 (13)	0.0037 (12)	−0.0031 (12)
C4	0.0216 (18)	0.0201 (17)	0.0117 (13)	−0.0037 (14)	0.0006 (12)	−0.0027 (12)
C5	0.0159 (16)	0.0180 (17)	0.0127 (14)	−0.0021 (13)	−0.0009 (12)	0.0028 (12)
C6	0.0123 (15)	0.0138 (15)	0.0148 (14)	−0.0024 (13)	0.0020 (11)	0.0025 (11)
C7	0.0150 (15)	0.0151 (16)	0.0160 (14)	0.0002 (13)	0.0029 (12)	0.0002 (12)
C8	0.0126 (16)	0.0118 (15)	0.0204 (16)	0.0002 (12)	0.0017 (12)	0.0001 (12)
C9	0.0103 (15)	0.0100 (14)	0.0190 (14)	0.0026 (12)	−0.0021 (11)	−0.0010 (11)
C10	0.0160 (16)	0.0127 (15)	0.0212 (15)	0.0007 (14)	−0.0074 (14)	−0.0018 (11)
C11	0.0226 (17)	0.0200 (17)	0.0154 (14)	0.0049 (14)	−0.0060 (12)	−0.0026 (13)
C12	0.0194 (17)	0.0177 (16)	0.0122 (13)	0.0032 (14)	−0.0009 (12)	0.0019 (11)
C13	0.0132 (15)	0.0121 (14)	0.0142 (13)	0.0041 (13)	0.0001 (12)	0.0012 (11)
C14	0.0110 (15)	0.0142 (16)	0.0191 (15)	0.0044 (13)	0.0012 (12)	−0.0013 (12)
Ni2	0.01145 (18)	0.01053 (18)	0.01407 (17)	−0.00027 (16)	−0.00135 (15)	0.00039 (13)
O1W	0.0169 (12)	0.0177 (12)	0.0231 (11)	−0.0015 (9)	−0.0018 (9)	0.0056 (9)
O2W	0.0143 (10)	0.0127 (11)	0.0247 (11)	0.0001 (9)	−0.0021 (8)	0.0009 (9)
N3	0.0191 (15)	0.0190 (15)	0.0195 (13)	−0.0019 (12)	−0.0024 (10)	0.0010 (11)
N4	0.0147 (13)	0.0142 (14)	0.0232 (13)	0.0001 (11)	−0.0017 (10)	−0.0007 (11)
N5	0.0148 (14)	0.0166 (13)	0.0174 (12)	−0.0005 (11)	−0.0015 (10)	0.0031 (10)
N6	0.0156 (13)	0.0147 (13)	0.0207 (14)	0.0001 (10)	0.0007 (10)	0.0028 (11)
C15	0.0248 (18)	0.0260 (19)	0.0201 (16)	0.0000 (15)	−0.0007 (13)	−0.0062 (14)
C16	0.0199 (19)	0.0188 (18)	0.0292 (17)	0.0016 (14)	−0.0029 (13)	−0.0074 (14)
C17	0.0163 (16)	0.0118 (15)	0.0350 (17)	0.0008 (14)	0.0018 (15)	0.0018 (12)
C18	0.0204 (18)	0.0197 (18)	0.0301 (18)	−0.0005 (14)	0.0004 (14)	0.0097 (14)
C19	0.0228 (19)	0.0250 (19)	0.0213 (15)	−0.0020 (15)	0.0025 (14)	0.0091 (13)
C20	0.025 (2)	0.0221 (18)	0.0179 (15)	0.0019 (14)	0.0001 (13)	−0.0043 (13)
C21	0.0234 (17)	0.0136 (15)	0.0252 (16)	−0.0018 (16)	0.0012 (16)	−0.0046 (12)

### Geometric parameters (Å, º)

**Table:**

Ni1—O1	2.099 (2)	Ni2—N3	2.074 (2)
Ni1—O3	2.109 (2)	Ni2—N4	2.088 (2)
Ni1—O5	2.111 (2)	Ni2—N5	2.095 (2)
Ni1—O7	2.1343 (19)	Ni2—N6	2.089 (3)
Ni1—N1	1.961 (2)	O1W—H1WA	0.8563
Ni1—N2	1.969 (2)	O1W—H1WB	0.8593
O1—C1	1.264 (4)	O2W—H2WA	0.8743
O3—C7	1.294 (3)	O2W—H2WB	0.8744
O2—C1	1.251 (4)	N3—H3A	0.9100
O4—C7	1.221 (4)	N3—H3B	0.9100
O5—C8	1.262 (3)	N3—C15	1.473 (4)
O7—C14	1.282 (3)	N4—H4A	1.0000
O6—C8	1.245 (3)	N4—C16	1.475 (4)
O8—C14	1.235 (4)	N4—C17	1.483 (4)
N1—C2	1.334 (4)	N5—H5A	1.0000
N1—C6	1.336 (4)	N5—C19	1.477 (4)
N2—C9	1.323 (4)	N5—C20	1.471 (4)
N2—C13	1.343 (4)	N6—H6A	0.9100
C1—C2	1.517 (4)	N6—H6B	0.9100
C2—C3	1.385 (4)	N6—C21	1.472 (4)
C3—H3	0.9500	C15—H15A	0.9900
C3—C4	1.396 (4)	C15—H15B	0.9900
C4—H4	0.9500	C15—C16	1.515 (5)
C4—C5	1.386 (4)	C16—H16A	0.9900
C5—H5	0.9500	C16—H16B	0.9900
C5—C6	1.382 (4)	C17—H17A	0.9900
C6—C7	1.523 (4)	C17—H17B	0.9900
C8—C9	1.524 (4)	C17—C18	1.509 (4)
C9—C10	1.391 (4)	C18—H18A	0.9900
C10—H10	0.9500	C18—H18B	0.9900
C10—C11	1.384 (4)	C18—C19	1.517 (5)
C11—H11	0.9500	C19—H19A	0.9900
C11—C12	1.396 (5)	C19—H19B	0.9900
C12—H12	0.9500	C20—H20A	0.9900
C12—C13	1.387 (4)	C20—H20B	0.9900
C13—C14	1.512 (4)	C20—C21	1.515 (4)
Ni2—O1W	2.131 (2)	C21—H21A	0.9900
Ni2—O2W	2.124 (2)	C21—H21B	0.9900
			
O1—Ni1—O3	156.79 (8)	N5—Ni2—O1W	93.07 (9)
O1—Ni1—O5	95.74 (8)	N5—Ni2—O2W	88.86 (9)
O1—Ni1—O7	89.96 (8)	N6—Ni2—O1W	87.13 (9)
O3—Ni1—O5	89.36 (8)	N6—Ni2—O2W	88.34 (9)
O3—Ni1—O7	94.68 (8)	N6—Ni2—N5	84.36 (10)
O5—Ni1—O7	155.62 (7)	Ni2—O1W—H1WA	121.7
N1—Ni1—O1	78.63 (9)	Ni2—O1W—H1WB	107.9
N1—Ni1—O3	78.19 (9)	H1WA—O1W—H1WB	116.6
N1—Ni1—O5	105.53 (9)	Ni2—O2W—H2WA	123.4
N1—Ni1—O7	98.84 (9)	Ni2—O2W—H2WB	116.2
N1—Ni1—N2	176.06 (10)	H2WA—O2W—H2WB	107.9
N2—Ni1—O1	99.61 (9)	Ni2—N3—H3A	110.5
N2—Ni1—O3	103.60 (9)	Ni2—N3—H3B	110.5
N2—Ni1—O5	78.10 (9)	H3A—N3—H3B	108.7
N2—Ni1—O7	77.58 (9)	C15—N3—Ni2	106.06 (18)
C1—O1—Ni1	114.33 (19)	C15—N3—H3A	110.5
C7—O3—Ni1	115.29 (18)	C15—N3—H3B	110.5
C8—O5—Ni1	115.02 (18)	Ni2—N4—H4A	106.6
C14—O7—Ni1	115.00 (19)	C16—N4—Ni2	107.41 (19)
C2—N1—Ni1	118.40 (19)	C16—N4—H4A	106.6
C2—N1—C6	122.0 (3)	C16—N4—C17	112.9 (2)
C6—N1—Ni1	119.5 (2)	C17—N4—Ni2	116.20 (19)
C9—N2—Ni1	118.88 (19)	C17—N4—H4A	106.6
C9—N2—C13	122.1 (2)	Ni2—N5—H5A	107.9
C13—N2—Ni1	119.07 (19)	C19—N5—Ni2	115.30 (19)
O1—C1—C2	115.9 (3)	C19—N5—H5A	107.9
O2—C1—O1	125.6 (3)	C20—N5—Ni2	106.17 (18)
O2—C1—C2	118.5 (3)	C20—N5—H5A	107.9
N1—C2—C1	112.2 (2)	C20—N5—C19	111.5 (2)
N1—C2—C3	120.6 (3)	Ni2—N6—H6A	110.4
C3—C2—C1	127.2 (3)	Ni2—N6—H6B	110.4
C2—C3—H3	120.9	H6A—N6—H6B	108.6
C2—C3—C4	118.1 (3)	C21—N6—Ni2	106.50 (18)
C4—C3—H3	120.9	C21—N6—H6A	110.4
C3—C4—H4	119.9	C21—N6—H6B	110.4
C5—C4—C3	120.2 (3)	N3—C15—H15A	110.1
C5—C4—H4	119.9	N3—C15—H15B	110.1
C4—C5—H5	120.8	N3—C15—C16	108.1 (3)
C6—C5—C4	118.4 (3)	H15A—C15—H15B	108.4
C6—C5—H5	120.8	C16—C15—H15A	110.1
N1—C6—C5	120.6 (3)	C16—C15—H15B	110.1
N1—C6—C7	112.8 (2)	N4—C16—C15	109.7 (3)
C5—C6—C7	126.6 (3)	N4—C16—H16A	109.7
O3—C7—C6	114.0 (3)	N4—C16—H16B	109.7
O4—C7—O3	126.7 (3)	C15—C16—H16A	109.7
O4—C7—C6	119.3 (3)	C15—C16—H16B	109.7
O5—C8—C9	115.1 (2)	H16A—C16—H16B	108.2
O6—C8—O5	126.8 (3)	N4—C17—H17A	109.0
O6—C8—C9	118.0 (3)	N4—C17—H17B	109.0
N2—C9—C8	112.7 (2)	N4—C17—C18	112.8 (3)
N2—C9—C10	120.9 (3)	H17A—C17—H17B	107.8
C10—C9—C8	126.4 (3)	C18—C17—H17A	109.0
C9—C10—H10	120.8	C18—C17—H17B	109.0
C11—C10—C9	118.3 (3)	C17—C18—H18A	108.2
C11—C10—H10	120.8	C17—C18—H18B	108.2
C10—C11—H11	119.9	C17—C18—C19	116.3 (3)
C10—C11—C12	120.1 (3)	H18A—C18—H18B	107.4
C12—C11—H11	119.9	C19—C18—H18A	108.2
C11—C12—H12	120.8	C19—C18—H18B	108.2
C13—C12—C11	118.4 (3)	N5—C19—C18	112.9 (3)
C13—C12—H12	120.8	N5—C19—H19A	109.0
N2—C13—C12	120.2 (3)	N5—C19—H19B	109.0
N2—C13—C14	113.4 (2)	C18—C19—H19A	109.0
C12—C13—C14	126.4 (3)	C18—C19—H19B	109.0
O7—C14—C13	114.2 (3)	H19A—C19—H19B	107.8
O8—C14—O7	126.3 (3)	N5—C20—H20A	109.9
O8—C14—C13	119.5 (3)	N5—C20—H20B	109.9
O2W—Ni2—O1W	174.88 (8)	N5—C20—C21	109.1 (2)
N3—Ni2—O1W	86.26 (9)	H20A—C20—H20B	108.3
N3—Ni2—O2W	92.25 (9)	C21—C20—H20A	109.9
N3—Ni2—N4	84.10 (10)	C21—C20—H20B	109.9
N3—Ni2—N5	174.54 (10)	N6—C21—C20	109.4 (2)
N3—Ni2—N6	101.01 (10)	N6—C21—H21A	109.8
N4—Ni2—O1W	87.83 (9)	N6—C21—H21B	109.8
N4—Ni2—O2W	96.90 (9)	C20—C21—H21A	109.8
N4—Ni2—N5	90.45 (10)	C20—C21—H21B	109.8
N4—Ni2—N6	172.57 (10)	H21A—C21—H21B	108.2
			
Ni1—O1—C1—O2	175.1 (2)	C2—C3—C4—C5	3.0 (4)
Ni1—O1—C1—C2	−6.2 (3)	C3—C4—C5—C6	−0.7 (4)
Ni1—O3—C7—O4	175.0 (3)	C4—C5—C6—N1	−1.7 (4)
Ni1—O3—C7—C6	−5.5 (3)	C4—C5—C6—C7	177.3 (3)
Ni1—O5—C8—O6	179.7 (2)	C5—C6—C7—O3	−174.5 (3)
Ni1—O5—C8—C9	−2.3 (3)	C5—C6—C7—O4	5.0 (5)
Ni1—O7—C14—O8	171.5 (2)	C6—N1—C2—C1	−179.3 (2)
Ni1—O7—C14—C13	−10.1 (3)	C6—N1—C2—C3	0.7 (4)
Ni1—N1—C2—C1	4.6 (3)	C8—C9—C10—C11	178.3 (3)
Ni1—N1—C2—C3	−175.4 (2)	C9—N2—C13—C12	−0.7 (4)
Ni1—N1—C6—C5	177.8 (2)	C9—N2—C13—C14	−178.9 (3)
Ni1—N1—C6—C7	−1.4 (3)	C9—C10—C11—C12	−0.4 (4)
Ni1—N2—C9—C8	2.9 (3)	C10—C11—C12—C13	0.6 (5)
Ni1—N2—C9—C10	−178.3 (2)	C11—C12—C13—N2	−0.1 (4)
Ni1—N2—C13—C12	178.5 (2)	C11—C12—C13—C14	177.9 (3)
Ni1—N2—C13—C14	0.3 (3)	C12—C13—C14—O7	−171.3 (3)
O1—C1—C2—N1	1.4 (4)	C12—C13—C14—O8	7.2 (5)
O1—C1—C2—C3	−178.6 (3)	C13—N2—C9—C8	−177.9 (2)
O2—C1—C2—N1	−179.8 (3)	C13—N2—C9—C10	1.0 (4)
O2—C1—C2—C3	0.2 (5)	Ni2—N3—C15—C16	45.9 (3)
O5—C8—C9—N2	−0.2 (4)	Ni2—N4—C16—C15	34.9 (3)
O5—C8—C9—C10	−178.9 (3)	Ni2—N4—C17—C18	−59.1 (3)
O6—C8—C9—N2	178.0 (3)	Ni2—N5—C19—C18	60.4 (3)
O6—C8—C9—C10	−0.8 (4)	Ni2—N5—C20—C21	−41.5 (3)
N1—C2—C3—C4	−3.0 (4)	Ni2—N6—C21—C20	−40.1 (3)
N1—C6—C7—O3	4.6 (4)	N3—C15—C16—N4	−55.3 (3)
N1—C6—C7—O4	−175.8 (3)	N4—C17—C18—C19	65.8 (4)
N2—C9—C10—C11	−0.4 (4)	N5—C20—C21—N6	56.4 (3)
N2—C13—C14—O7	6.8 (4)	C16—N4—C17—C18	176.1 (3)
N2—C13—C14—O8	−174.7 (3)	C17—N4—C16—C15	164.3 (3)
C1—C2—C3—C4	177.0 (3)	C17—C18—C19—N5	−67.0 (4)
C2—N1—C6—C5	1.8 (4)	C19—N5—C20—C21	−167.8 (3)
C2—N1—C6—C7	−177.4 (3)	C20—N5—C19—C18	−178.4 (3)

### Hydrogen-bond geometry (Å, º)

**Table:**

*D*—H···*A*	*D*—H	H···*A*	*D*···*A*	*D*—H···*A*
N3—H3*A*···O8^i^	0.91	2.41	3.213 (3)	147
N3—H3*B*···O4^ii^	0.91	2.11	3.015 (3)	176
N4—H4*A*···O1	1.00	2.07	3.054 (3)	167
N5—H5*A*···O2	1.00	2.08	3.054 (3)	163
N6—H6*A*···O3^ii^	0.91	2.14	2.986 (3)	154
N6—H6*B*···O6^iii^	0.91	2.07	2.943 (3)	160
O1*W*—H1*WA*···O1	0.86	2.56	3.088 (3)	121
O1*W*—H1*WA*···O2	0.86	2.00	2.795 (3)	154
O1*W*—H1*WB*···O3^ii^	0.86	1.91	2.757 (3)	170
O2*W*—H2*WA*···O7^i^	0.87	1.80	2.663 (3)	169
O2*W*—H2*WB*···O6^iii^	0.87	1.90	2.742 (3)	160

Symmetry codes: (i) *x*−1, *y*, *z*; (ii) −*x*+2, *y*+1/2, −*z*+3/2; (iii) −*x*+1, *y*+1/2, −*z*+3/2.
